# Alcoholysis kinetics and mechanism studies of ethyl levulinate production from ball milled corn stover

**DOI:** 10.1039/d2ra05644e

**Published:** 2022-11-29

**Authors:** Huan Liu, Haibo Meng, Hongbin Cong, Xiuli Shen, Xueli Chen, Haohan Xing, Jinhang Dai

**Affiliations:** Academy of Agricultural Planning and Engineering, Key Laboratory of Energy Resource Utilization from Agriculture Residue, Ministry of Agriculture and Rural Affairs Beijing 100125 China liuhuan19851208@163.com conghongbin@163.com shenxiuli111@163.com xhh523059864@126.com +86-10-59196858 +86-10-59196858; Department of Agricultural and Biological Engineering, Purdue University West Lafayette IN 47907 USA xuelic@hotmail.com; College of Environment and Resources, Chongqing Technology and Business University Chongqing 400067 China nhangdai@ctbu.edu.cn

## Abstract

Alcoholysis of ball-milled biomass over catalysts with Brønsted and Lewis acid sites provides an efficient and sustainable scheme to produce versatile biobased chemicals under mild conditions; however, optimizing the process parameters is challenged by the complexity of reaction pathways and the multiplicity of ball milling and combination catalyst gains. To address these challenges, we present kinetic analysis of ethyl levulinate (EL) production from ball-milled corn stover catalyzed by Brønsted (B) acidic ionic liquid [Bmim-SO_3_H][HSO_4_] (SO_3_H-IL) and Lewis (L) acidic Al_2_(SO_4_)_3_. Product analysis shows that cellulosic substrates can form EL either through the intermediate ethyl-d-glycopyranoside (EDGP) or levoglucosenone (LGO), with the former leading the alcoholysis reaction. Kinetics results reveal that ball milling accelerates the reaction rate by promoting the formation of EDGP and LGO from cellulose. Pure SO_3_H-IL gives high selectivity towards EDGP from ball-milled corn stover and promotes the LGO production, whereas addition of Al_2_(SO_4_)_3_ substantially facilitates their further conversion to EL. Our findings contribute to the rational design of efficient catalytic strategies for sustainable and profitable biorefinery.

## Abstract 2

Kinetic analysis of ethyl levulinate (EL) production from ball-milled corn stover catalyzed by Brønsted (B) acidic ionic liquid [Bmim-SO_3_H][HSO_4_] (SO_3_H-IL) and Lewis (L) acidic Al_2_(SO_4_)_3_ was performed in this study.

## Graphical abstract

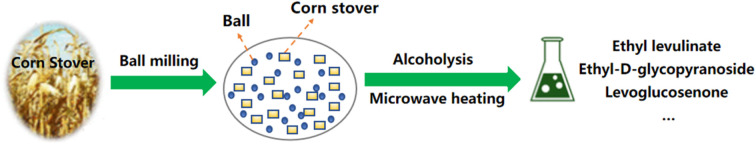

## 1. Introduction

With the gradual depletion of fossil resources and rising environmental concerns, increasing effort has been devoted to exploring renewable resources. Lignocellulosic biomass is regarded as an ideal substitute for fossil resources, which can be transformed into fuels and chemicals through a variety of processing methods.^1–3^ Alcoholysis technology for converting lignocellulose to biobased chemicals including alkyl glucopyranoside, alkyl levulinates, levoglucosenone (LGO), *etc.* has so far attracted considerable interest, with the advantage of depressing the formation of humins.^4–7^ Many researchers have reported the alcoholysis of biomass using various acid catalysts, such as corrosive sulfuric acid,^4,5,8^ costly noble-metal catalysts^9^ and heterogeneous catalysts like sulfated metal oxides, zeolite, and heteropoly acids.^6^ Among them, some scholars reported the yield of levulinate esters production using sulfuric acid as catalyst. Garves investigated the degradation of cellulose by alcohols and sulfuric acid catalysts, and the yield of 44.0% was obtained.^4^ Chang *et al.* studied the catalytic conversion of wheat straw in the presence of sulfuric acid, the yield of ethyl levulinate 51.0% was obtained.^7^ Le Van Mao *et al.* reported the yield of ethyl levulinate 44.4% from pine wood with sulfuric acid as catalyst.^8^ In addition, Saravanamurugan reported catalytic transformation of the sugars with sulfonic acid functionalized cation ([Bmim-SO_3_H] and [NEt_3_B-SO_3_H]) ionic liquids as catalyst, ethyl levulinate was found the yield ranging from 67% to 77% from fructose.^10^ Several studies revealed that the mixed-acids consisting of both Brønsted (B) and Lewis (L) acids showed a higher efficiency on the conversion of glucose.^11,12^ B acid catalysts are effective for the dehydration of carbohydrates with alcohol as both solvent and reactant,^8^ meanwhile, L acid catalysts show higher catalytic activity for the isomerization of glucose to fructose as compared with B acid.^13^ Under the same conditions, fructose will be more likely to be converted to levulinate esters than glucose.^9^ However, fructose is less abundant and more expensive than glucose. Therefore, L acid could be introduced into the catalytic system to make glucose isomerization into fructose, so as to promote the occurrence of the reaction. Al_2_(SO_4_)_3_, containing both L and B acid sites, have been shown to be efficient for such conversions.^14^ Recently, our previous investigation found that the combined catalyst system with a strong B acidic ionic liquid [BmimSO_3_H][HSO_4_] (SO_3_H-IL) and a L acidic Al_2_(SO_4_)_3_ was efficient in biomass carbohydrate conversion.^15^ In order to developed a deeper understanding of alcoholysis mechanism and provide in-depth study about the B + L catalytic system influence on the conversion of lignocellulose. It's attractive to establish a further detailed study on the combination B + L catalysed alcoholysis of corn stover.

The kinetic model is important and significant due to it can be not only contributed to optimize the process conditions, but also to understand the reaction rule and scale up the reaction process in industry.^16^ The kinetic parameters could provide useful information for understanding the alcoholysis process. Currently, there have many studies focused on the kinetics of lignocellulosic biomass hydrolysis to levulinic acid.^17–20^ However, the kinetic model describing the acid-catalyzed alcoholysis of lignocellulosic biomass into levulinate esters, including the formation of by-products, is lacking. Peng *et al.*^21^ explored the kinetic investigation on the synthesis of methyl levulinate from glucose in methanol medium by extremely low sulfuric acid. The reported study developed a simplified kinetic model of first-order reaction for methyl levulinate formation from methyl glucosides, and a single B acid sulfuric acid was used as catalyst in this kinetic study. More recently, Chen *et al.*^22^ conducted a kinetic study on acid-catalyzed alcoholysis of ball-milled corn stover to produce ethyl levulinate, and a single sulfuric acid was used as catalyst. To our best knowledge, the kinetic study on the alcoholysis reaction by using both B and L acid as catalysts is poorly understood.

Apart from that, lignocellulosic biomass is composed mostly of the biopolymers cellulose, hemicellulose and lignin, along with small amounts of extractives.^23,24^ However, the complexity of cell wall constituents and structural heterogeneity of lignocellulosic plant cell wall and tissues association are mainly responsible for the lignocellulose recalcitrance, hindering its high-value utilization.^25^ The major technical challenge for the effective utilization of lignocellulose fractions in the bio-refinery is overcoming biomass recalcitrance. To overcome recalcitrance, various pretreatment methods, involving physical, alkaline, acid, oxidative and solvent processes have been extensively studied.^26^ Conventional ball-milling is a predominately physical process, which can reduce the particle size of biomass material and destroy the matrix of the tissue and cell walls, and increase enzymatic hydrolysis efficiency, meanwhile, it produces no hydrolysis or fermentation inhibitors.^27^ Our previous work showed that ball milling is not only a mechanical pulverization process, but it can also cause a mechanical–chemical effect due to the depolymerization of macromolecule in the cell wall during grinding.^28–30^ Fragmentation of biomass is always accompanied by modifications in its physicochemical properties, favoring further biological or chemical degradation. Previous studies were mainly concerned about the structural characteristics of lignocellulose after mechanical deconstruction and its contribution to enzymatic hydrolysis.^27,31,32^ Few studies have focused on the application of mechanical depolymerization on acid-catalyzed alcoholysis.

In this study, to gain insights into the alcoholysis process for the conversion of ball milled corn stover using combination catalysts of SO_3_H-IL and Al_2_(SO_4_)_3_, we conducted a kinetic study on the acid-catalyzed alcoholysis of ball-milled corn stover to cellulose-derived product, *i.e.*, EDGP, LGO, EL, with emphasis on alcoholysis mechanism of lignocellulose. In order to optimize the utilization of lignocellulosic materials, a greater scientific understanding on the kinetic parameter is of vital importance to understand the mechanisms of mechanical force applied in the subsequent lignocellulosic bioconversion process and the combined B + L catalyst system on the alcoholysis, and the factors that can influence the performances of alcoholysis and conversion.

## 2. Materials and methods

### 2.1 Materials preparation

Corn stover was collected in Shangzhuang experiment station of China Agriculture University (Beijing, China). The raw material was air-dried to the moisture content of 4.16% and first roughly cut into less than 2 cm. The carbohydrates and lignin contents of biomass samples were determined using NREL methods.^33^ The chemical composition of corn stover (based on a dry basis) was 33.20% glucan, 22.34% xylan, 4.02% arabinan and 16.49% lignin.

### 2.2 Chemicals

EL, LGO, ethyl-α-d-glycopyranoside (E-αDGP), ethyl-β-d-glycopyranoside (E-βDGP) were obtained from TCI (Shanghai). 5-Ethoxymethylfurfural (5-EMF) and glucose (Glu) were the analytical grade from Sigma Aldrich (St. Louis, MO, USA). Al_2_(SO_4_)_3_·18H_2_O metal salt catalyst was purchased from Sinopharm Chemical Reagent Factory, China.

### 2.3 Mechanical ball-milling pretreatment of corn stover

Corn stover was pretreated by mechanical grinding before alcoholysis. Corn stover was milled in an RT-34 hammer mill (Hongquan Pharmaceutical Machinery Ltd, China). The coarsely milled material was obtained by passing through a 40-mesh screen, denoted as CM. The ball milled sample was obtained by further milling CM sample in a vibration grind mill CJM-SY-B (Qinhuangdao Taiji Ring Nano Ltd, Hebei, China), mixed with ZrO_2_ balls (6–10 mm diameter) in a volume ratio of 1 : 2 for 60 min. The ball milled sample was denoted BM. The ball milling process was controlled below 30 °C by a cooling water system.

### 2.4 Alcoholysis reaction

#### 2.4.1 Synthesis of B acidic ionic liquid

SO_3_H-functionalized Brønsted(B) acidic ionic liquid [Bmim-SO_3_H][HSO_4_] (SO_3_H-IL) catalyst was synthesized according to the procedure previously reported.^10^ The identity and purity of the Brønsted acidic ionic liquid [Bmim-SO_3_H] [HSO_4_] was confirmed by NMR. ^1^H-NMR and ^13^C-NMR spectra were recorded on a JOEL JNM-EcA600 NMR spectrometer in D_2_O at 25 °C. ^1^H NMR (600 MHz, D_2_O): *δ*/ppm = 1.54–1.62 (m, 2H; CH_2_), 1.82–1.90 (m, 2H; CH_2_), 2.75–2.84 (t, 2H; CH_2_–SO_3_H), 3.70 (S, 3H; N–CH_3_), 4.01–4.14 (m, 2H; CH_2_), 7.25 (S, 1H; CH), 7.31 (S, 1H; CH), 8.54 (S, 1H; N–CH–N); ^13^C NMR (600 MHz, D_2_O): *δ*/ppm = 20.9, 28.1, 35.6, 48.8, 50.1, 122.1, 123.7, 136.0.

#### 2.4.2 Alcoholysis reaction of biomass carbohydrates and product analysis

Alcoholysis reaction was performed in a Milestone microwave lab station (Italy). Alcoholysis reactants such as corn stover (1 g), solvent ethanol (20 mL), and a given amount of catalyst were introduced into 100 mL sealed Teflon tube reaction vessels. The reaction system is equipped with a stirring device with stirring bar. After pre-stirring for 1 min, the sample was heated in the microwave reactor to the desired temperature for a specified reaction time while being vigorously stirred. After the reaction, the vessel was quenched in an ice water bath to terminate the reaction.

#### 2.4.3 Product analysis

EL and LGO were analyzed by gas chromatography (Shimadzu, Japan) equipped with a DB-5 capillary column (30 m × 0.25 mm, 0.25 μm) using a flame ionization detector (FID) operating at 250 °C. EDGP was measured by FID-equipped HPLC and the HPX-87H column. Aqueous sulfuric acid (5 mM) was used as the mobile phase with a flow of 0.6 mL min^−1^ at 80 °C. Calibration curves were established for quantitative calculations. The product yields were calculated on a molar basis according to the following formula:

1 Yields (mol%) = *C*_1_ × *M*_0_ × 100%/(*C*_0_ × *M*_1_)

where *C*_0_ is the initial mass of carbohydrates, *C*_1_ represents the mass of liquefaction product, and *M*_0_ and *M*_1_ denote the carbohydrate and product molecular weights, respectively.

## 3. Results and discussion

### 3.1 Alcoholysis reaction products and pathways

**Scheme 1:**
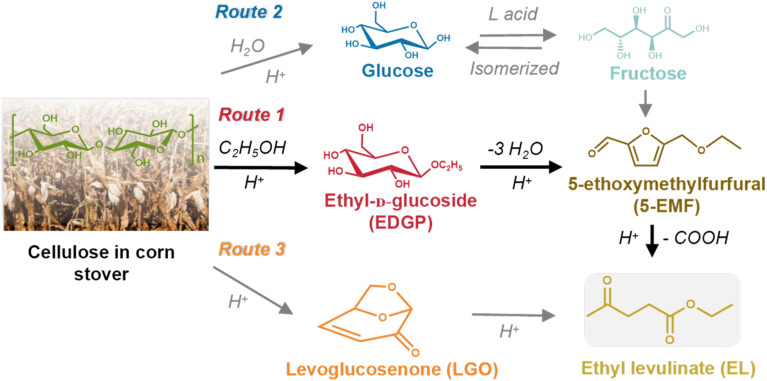
Roadmap for the conversion of cellulose in corn stover to EL in ethanol solvent with SO_3_H-IL and Al_2_(SO_4_)_3_ as catalyst.

The main target products and intermediates were quantitated by GC or HPLC to reveal the reaction pathway catalyzed by SO_3_H-IL and Al_2_(SO_4_)_3_. According to our previous work,^15^ the alcoholysis products of cellulose in corn stover were mainly ethyl levulinate (EL), intermediate Glu, E-αDGP, E-βDGP, henceforth, “EDGP”, together, 5-ethoxymethylfurfural (5-EMF), and levoglucosenone (LGO). A plausible reaction pathway for the acid-catalyzed decomposition of cellulose in ethanol medium is proposed in Scheme 1. Among all of the decomposition compounds, the intermediate EDGP and the final product EL existed in large quantities during the reaction process. Based on product analysis, the formation of EL is primarily from Route 1, which probably proceeds by reacting glucose unit in cellulose with ethanol to give EDGP, followed by dehydration to yield 5-EMF, then, hydration and esterification to form EL. Meanwhile, water was formed during the EL production and through the side reactions such as the self-condensation of ethanol. Huang *et al.*^34^ found that Al_2_(SO_4_)_3_ tended to hydrolyze with water to generate [Al(OH)_*x*_(H_2_O)_*y*_]^*n*+^ and H^+^, which were the actual Lewis and Brønsted acid sites that participated in the subreactions involved in cellulose conversion. In addition, the cellulose component in corn stover was hydrolyzed through Route 2 to form glucose, which could isomerize into fructose in the presence of Al_2_(SO_4_)_3_-containing L acid sites. Then, fructose dehydrated to produce 5-EMF, followed by the rapid formation of EL. During the alcoholysis reaction, a remarkable LGO was produced from the pyrolysis of cellulose. The “hot spots” phenomenon caused by microwave heating may occur in the reaction system, inducing the actual temperature in some regions to be much higher than the set temperature.^35^ In this case, LGO was generated from the pyrolysis of cellulose due to the high local temperature. This was confirmed in our previous study by the alcoholysis of LGO alone and the identification of EL as the major degradation product.^15^ Sarotti *et al.*^36^ also found that cellulose can be pyrolyzed under microwave irradiation to produce LGO. Therefore, LGO can be an important intermediate to yield EL as illustrated in Route 3.

### 3.2 Effects of process variables on the ethanolysis of cellulose in corn stover

Effects of different process parameters including variable temperature, ball milling pretreatment and catalyst type on the yields of products were studied as a function of reaction time. According to our previous study, among all of the decomposition compounds of cellulose, the intermediate EDGP and the final product EL existed in large quantities during the alcoholysis process. In addition, a remarkable amount of LGO was detected. LGO is generally produced by catalyzed pyrolysis of lignocellulosic biomass or cellulose at a temperature over 240 °C.^37^ In the microwave-assisted alcoholysis of corn stover, the LGO production may be ascribed to the specific thermal effects of microwave heating. The specific thermal effects of microwave cause the local high temperature “hot spot” phenomenon in the reaction mixture which can be generally observed on the solid–liquid interface in heterogenous reactions.^38^ In this reaction system, the hot spots phenomenon may occur at the phase boundaries between corn stover particles and the surrounding liquid solvent. The considerable LGO production may be contributed to the catalytic pyrolysis of cellulose mixed with ionic liquid. Kudo *et al.* reported the catalytic pyrolysis of cellulose by mixing with [BMMIM]CF_3_SO_4_(IL), forming levoglucosenone in high yield at 250 °C. It was reported that the role of the ionic liquids was to facilitate the formation of electron donor–acceptor (EDA) complexes with hydroxyl groups at the C6 position, leading to the opening of hydrogen bonds between the cellulose molecules, and finally the dissolution of cellulose.^39,40^ It is plausible that SO_3_H-IL in our reaction system acted as a catalyst during pyrolysis. The dissolved cellulose was highly exposed to B acid SO_3_H-IL, which could facilitate the dehydration reaction to yield a remarkable amount of LGO. Therefore, the target product EL as well as the main intermediate products EDGP and LGO were analyzed and quantified. Furthermore, the experiments without using catalyst were carried out on nonball-milled corn stover and ball-milled corn stover. The results showed that no intermediate was detected in the reaction system in the absence of catalyst.

#### 3.2.1 Effect of reaction temperature on alcoholysis of nonball-milled corn stover catalyzed by SO_3_H-IL

**Fig. 1:**
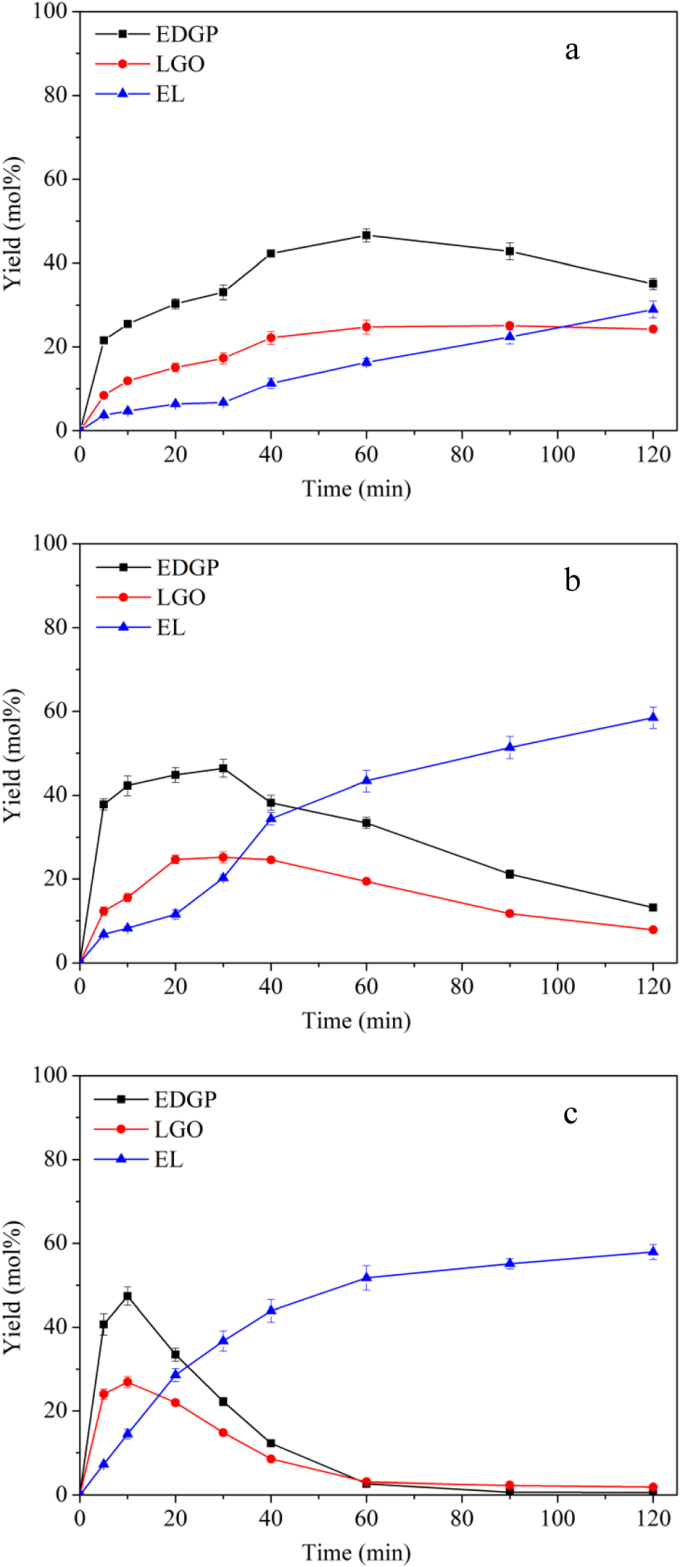
Effects of reaction temperature on conversion of nonball-milled corn stover catalyzed by SO_3_H-IL into the main chemicals. Reaction conditions: corn stover, 1 g; ethanol, 20 g; SO_3_H-IL, 2.0 mmol. The product yields were calculated based on the total content of glucan, xylan and arabynosyl residues. (a): 160 °C, (b): 170 °C, (c): 180 °C.

Reaction temperature is usually a particularly important parameter in the chemical reaction process, determining chemical reaction rate and conversion efficiency.^10^ Three different temperatures (160 °C, 170 °C, 180 °C) were applied for the conversion of nonball-milled corn stover to EL using SO_3_H-IL as acid catalyst. It can be observed that the yield of EDGP grew with temperature, reaching the maximum yield within 10 min at 180 °C (Fig. 1c). With the extension of alcoholysis time, the content of EDGP gradually decreased. It should be pointed out that the decomposition of EDGP takes place at a faster rate as the temperature increases. Additionally, a significant increase of EL can be seen with the prolonging of reaction time. These findings indicated that high temperatures promote the conversion of EDGP to EL. Moreover, in the early stage of alcoholysis, the yield of LGO increased accordingly with the augment of temperature. However, with the prolongation of reaction time, the elevation of temperature also promoted the degradation of LGO.

#### 3.2.2 Effect of reaction temperature on alcoholysis of ball-milled corn stover catalyzed by SO_3_H-IL

**Fig. 2:**
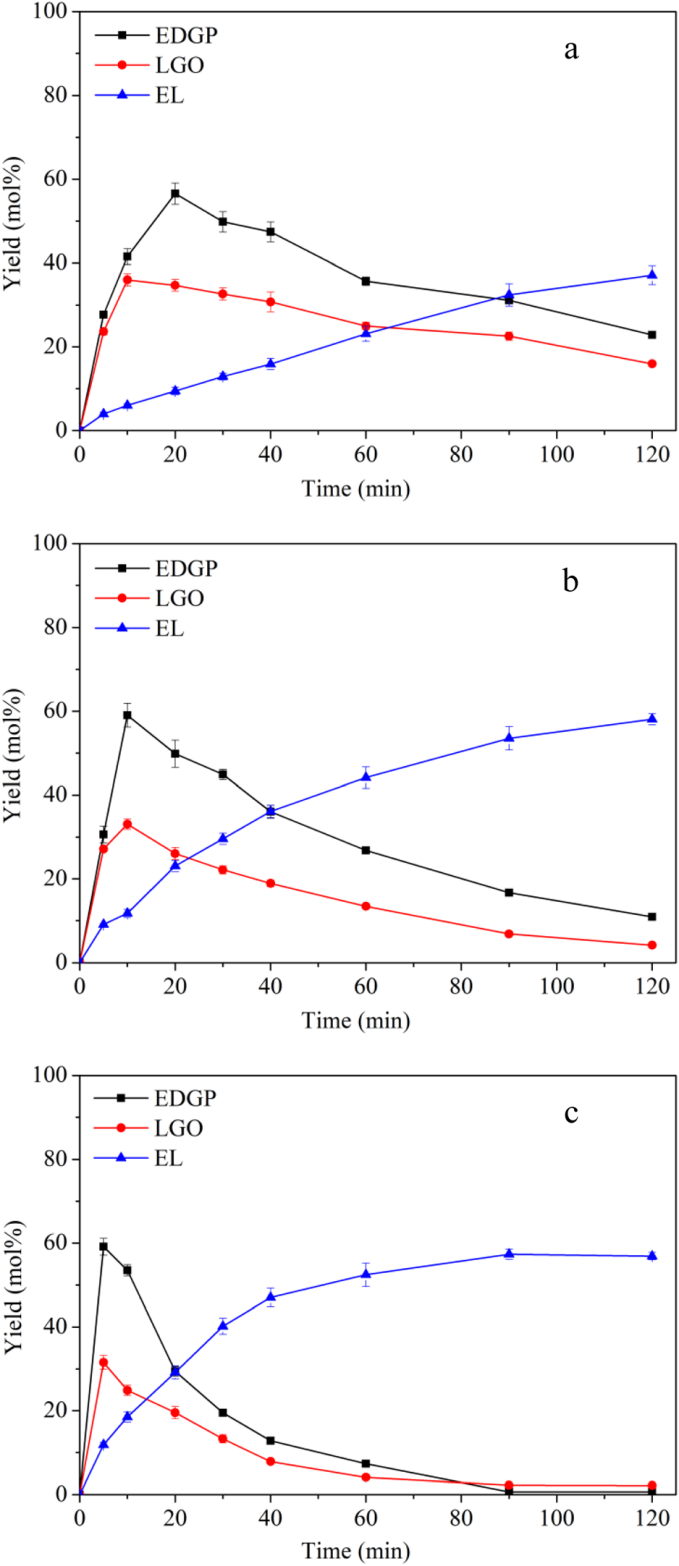
Effects of reaction temperature on conversion of ball-milled corn stover catalyzed by SO_3_H-IL into the main chemicals. Reaction conditions: corn stover, 1 g; ethanol, 20 g; SO_3_H-IL, 2.0 mmol. The product yields were calculated based on the total content of glucan, xylan and arabynosyl residues. (a): 160 °C, (b): 170 °C, (c): 180 °C.

The SO_3_H-IL catalyzed conversion of ball-milled corn stover to EL was then investigated. Fig. 2 illustrates the influence of different temperatures (160 °C, 170 °C, 180 °C) on the yields of intermediates and products over reaction time. Comparing with the nonball-milled corn stover, ball-milled corn stover achieved the maximum yield of EDGP earlier within relatively short time, and the maximum yields of EDGP were higher than those of nonball-milled sample. A possible explanation for ball milling promoting the synthesis of EDGP is that mechanical pretreatment could decompose the carbohydrate macromolecules in the cell wall, which could facilitate the alkylation of the glucose unit in cellulose to form EDGP. Interestingly, the same trend also occurred in the formation of LGO, with a maximum yield of 36.03 mol% at a lower temperature of 160 °C in Fig. 2a, indicating that mechanical pretreatment also promoted the pyrolysis of cellulose to form LGO. With the elevation of alcoholysis temperature, the yield of EDGP and LGO increased significantly. Normally, elevated temperature can contribute to the enhancement of reaction rate and conversion efficiency.^6^ As shown in Fig. 2c, the EDGP and LGO yield reached the maximum value within 5 min at the temperature of 180 °C. Subsequently, they were respectively fast-transformed to EL with the prolonging of alcoholysis time.

#### 3.2.3 Effect of reaction temperature on alcoholysis of ball-milled corn stover catalyzed over SO_3_H-IL combined with Al_2_(SO_4_)_3_

**Fig. 3:**
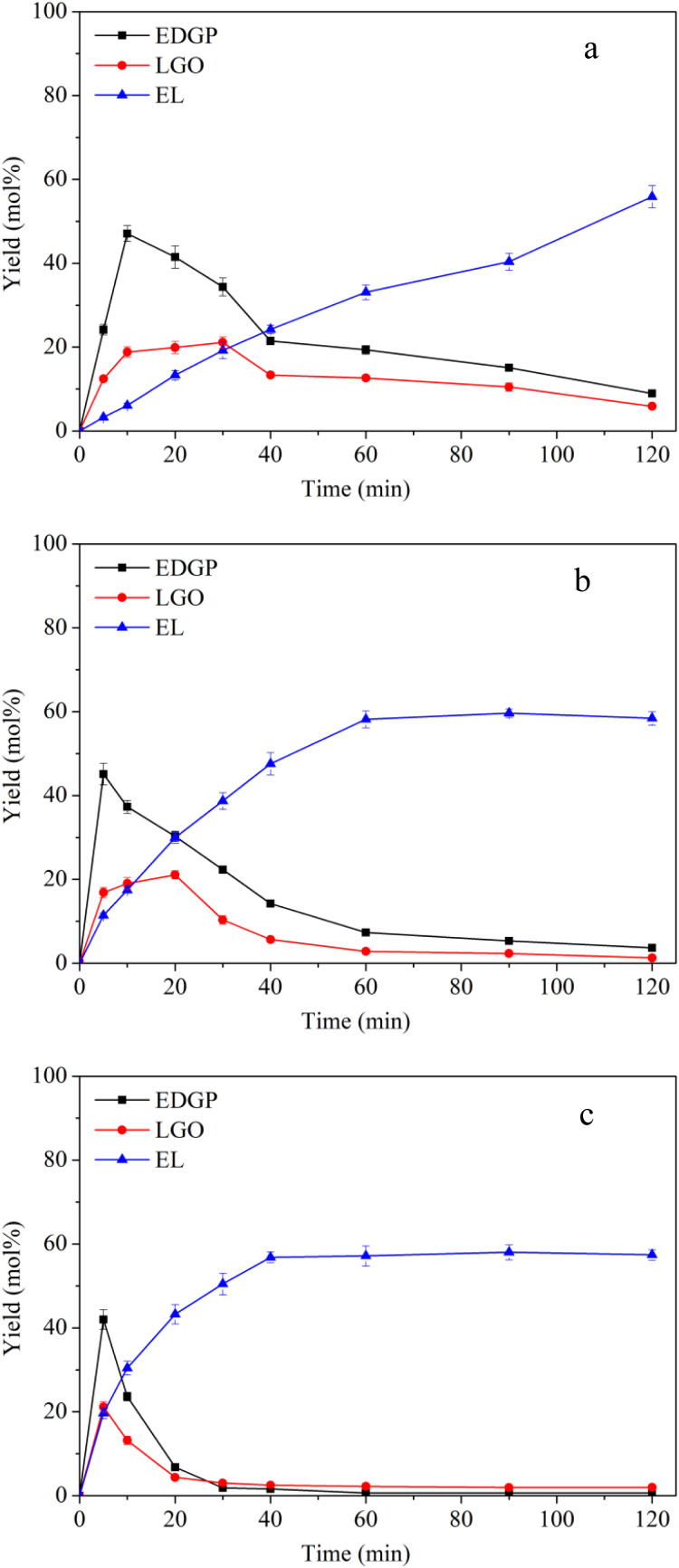
Effects of reaction temperature on conversion of ball-milled corn stover catalyzed by SO_3_H-IL and Al_2_(SO_4_)_3_ into the main chemicals. Reaction conditions: corn stover, 1 g; ethanol, 20 g; SO_3_H-IL, 1.6 mmol; Al_2_(SO_4_)_3_, 0.4 mmol. The product yields were calculated based on the total content of glucan, xylan and arabynosyl residues. (a): 160 °C, (b): 170 °C, (c): 180 °C.

To understand the B + L acid catalyzed degradation process of ball-milled corn stover to EL in ethanol medium, alcoholysis experiments were carried out using SO_3_H-IL and Al_2_(SO_4_)_3_ as the catalysts at reaction temperatures in a range of 160–180 °C. As shown in Fig. 3, the generation of EL was promoted when Al_2_(SO_4_)_3_ was introduced in the reaction. The yield of EL 55.88 mol% was obtained with B + L acid catalyzed at 160 °C in Fig. 3a, however, with pure B acid in Fig. 2a, the yield of EL was 37.14 mol%. Meanwhile, the yield of EDGP rose sharply to the maximum, which was lower than that catalyzed by the single B acid SO_3_H-IL. Subsequently, EDGP was rapidly converted to EL with the extension of alcoholysis time. The above results suggest that Al_2_(SO_4_)_3_ containing L acid sites was proposed to promote the aldose-ketose isomerization of glucose to fructose, could facilitate the EL formation. Moreover, Al_2_(SO_4_)_3_ containing Al^3+^ could combine with EDGP to form Al–EDGP complex. Zhang *et al.* have proposed that Al^3+^ and MDGP can assemble into tetradentate Al–MDGP complexes by using ESI-MS analysis. This coordination structure may change the electric charge distribution in the pyranose ring, which can induce the conversion of MDGP into 5-MMF.^41^ Therefore, we hypothesized that Al_2_(SO_4_)_3_ containing Al^3+^ could assemble with EDGP into a catalytic Al–EDGP complex, thus facilitating EDGP conversion in the current catalytic system. Additionally, the yield of LGO catalyzed by B + L acid in Fig. 3 was lower than that catalyzed by the single B acid SO_3_H-IL in Fig. 2. When SO_3_H-IL combined with Al_2_(SO_4_)_3_ was used as catalyst, the proportion of B acid decreased. The reduction in SO_3_H-IL content in the mixture resulted in a slight decrease in the yield of LGO, probably due to the less dissolved cellulose exposed to strong B acid SO_3_H-IL. Therefore, the lower B acid ratio with weaker dehydration capacity led to the reduction of LGO formation.

### 3.3 Development of kinetic models

**Scheme 2:**
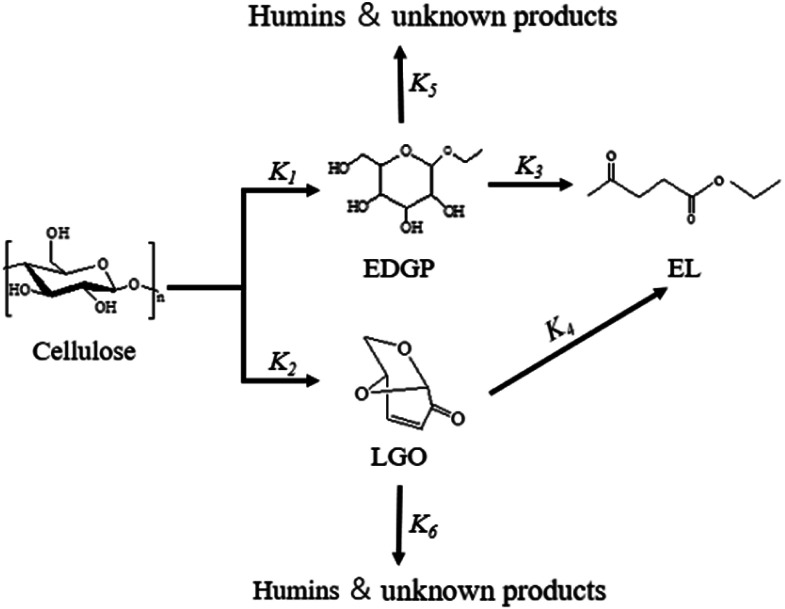
Simplified model for the conversion of cellulose in corn stover to EL in ethanol solvent with SO_3_H-IL and Al_2_(SO_4_)_3_ catalyst.

According to the product analysis, the intermediates EDGP and LGO, together with the final product EL were characterized as the three major compounds of corn stover ethanolysis. During the experiments, 5-EMF and glucose were detected in small amounts. In addition, some dark-brown insoluble substances known as humins were also observed. They are probably side reaction products of acid-catalyzed decompositions of the reactant and/or certain products under applied experimental conditions. Due to the instability of the low yield intermediates and the complexity of the reaction pathways, it is difficult to find a rigorous mechanism for investigating the formation of EL from cellulose in corn stover. Thus, a simplified model was used to study the kinetics of cellulose decomposition to EL as given in Scheme 2. The key reaction pathway in the alcoholysis reaction is the alkylation of cellulose to generate EDGP, followed by dehydration to yield 5-EMF, and finally esterification to form EL. Meanwhile, cellulose is degraded to LGO by pyrolysis, which was sequentially converted to generate EL as the final product.

Here, some assumptions according to the experimental results were made as follows: (1) the formation of intermediate products with low yield is negligible, *i.e.*, 5-EMF, glucose; (2) all the unknown products and humins are considered to be byproducts; (3) cellulose decomposes to EL and undesired byproducts in a parallel reaction mode. According to the above assumptions, cellulose decomposition to EL and by-products can be viewed as first-order reactions. A pseudo-homogeneous irreversible first-order reaction model on the formation of EL from cellulose is proposed as shown in Scheme 2. Based on the model, the concentrations of cellulose (CEL), EDGP, LGO and EL as a function of time can be presented as follows:2
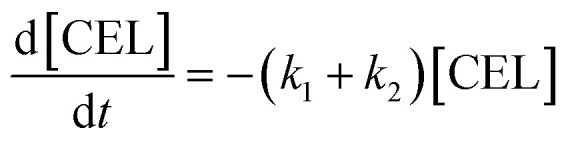
3
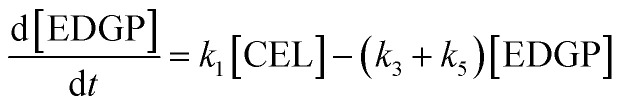
4
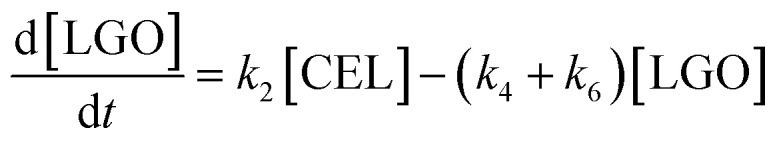
5
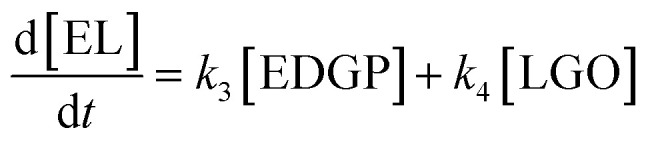


Solving the differential equations, the analytic expression of concentration of CEL, EDGP, LGO and EL can be obtained:

6 [CEL] = [CEL]_0_ exp[−(*k*_1_ + *k*_2_)*t*]

7
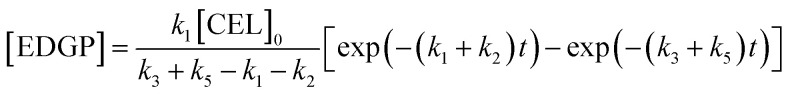
8
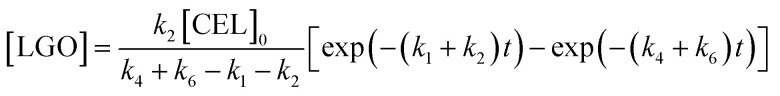
9
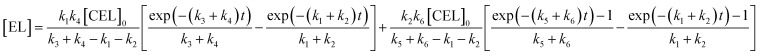
where [CEL], [EDGP], [LGO] and [EL] respectively represent the molar conversion rate of CEL (%), the yield of EDGP, LGO and EL (%). [CEL]_0_ is the initial yield of cellulose (100%). *k* is the reaction rate constant for cellulose degradation, EDGP or LGO conversion to EL and by-products.

**Fig. 4:**
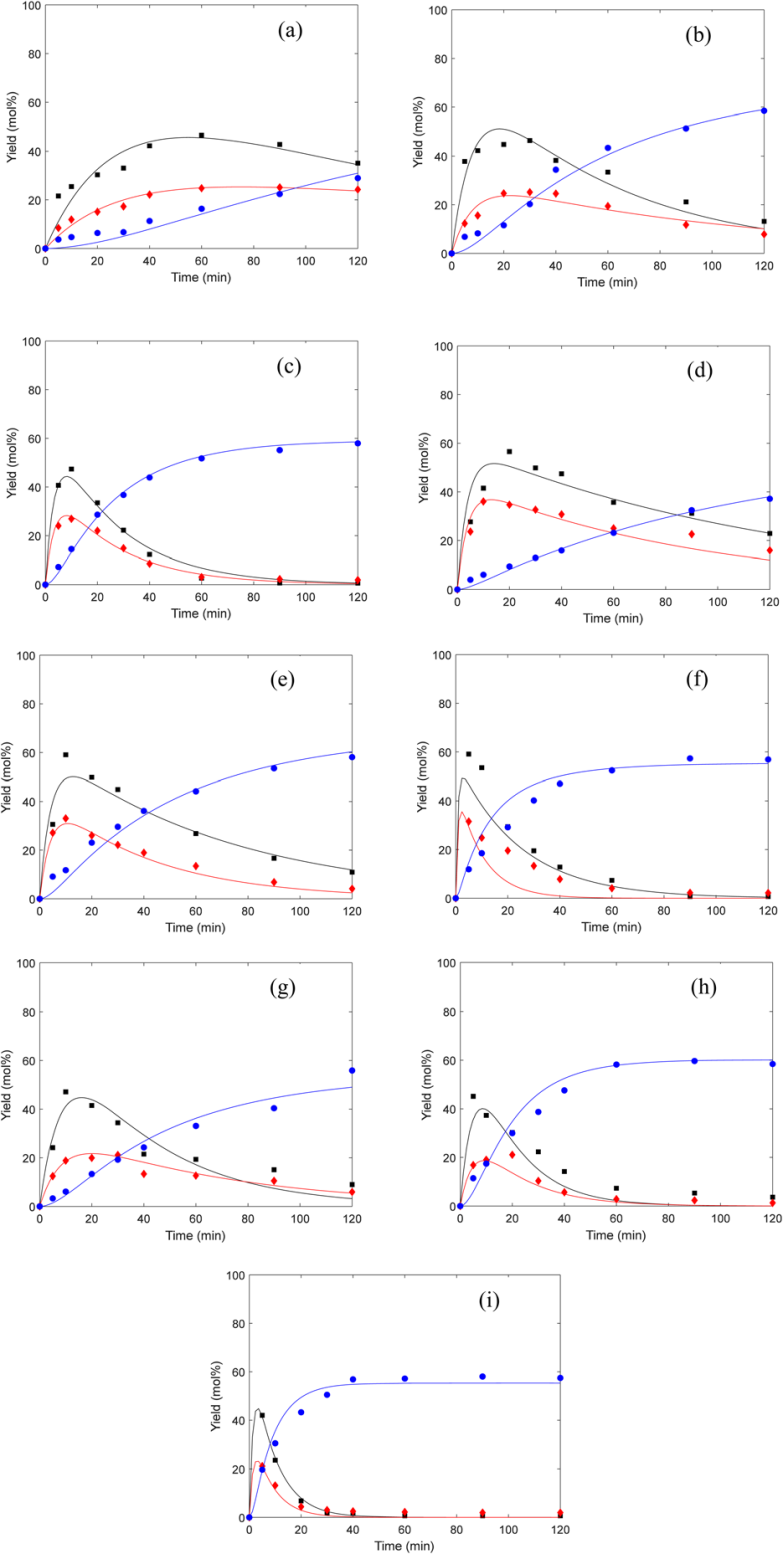
Comparison of experimental data (■: EDGP; ♦: LGO; ●: EL) and kinetic models (solid lines) (a): CM, B acid, 160 °C; (b): CM, B acid, 170 °C; (c): CM, B acid, 180 °C; (d): BM, B acid, 160 °C; (e): BM, B acid, 170 °C; (f): BM, B acid, 180 °C; (g): BM, B + L acid, 160 °C; (h): BM, B + L acid, 170 °C; (i): BM, B + L acid, 180 °C.

The kinetic parameters in the equations were estimated using the method of non-linear least squares regression analyses by MATLAB. Fig. 4 shows the curve fitting for the experimental data of the cellulose conversion to EL at temperatures of 160 °C, 170 °C and 180 °C. Table 1 summarizes the apparent reaction rate constants, as show in Table 1, high correlations (*R* > 0.8) between the experimental and predicted values were obtained, suggesting a good robustness of the model.

**Table 1:** Rate constants of cellulose decomposition with different raw materials and catalysts at various reaction temperatures

Raw material	Catalyst	Temp. (°C)	*k* (min^−1^)						*R* ^2^		
			*k*_1_	*k*_2_	*k*_3_	*k*_4_	*k*_5_	*k*_6_	*R*_1_^2^	*R*_2_^2^	*R*_3_^2^
CM	B acid	160	0.0249	0.0115	0.0067	0.0003	0.0006	0.0027	0.8767	0.9592	0.9099
		170	0.0877	0.0365	0.0163	0.0013	0.0012	0.0083	0.9283	0.9277	0.9770
		180	0.1709	0.1092	0.0361	0.0030	0.0031	0.0367	0.9855	0.9849	0.9960
BM	B acid	160	0.1469	0.1076	0.0032	0.0086	0.0048	0.0022	0.8657	0.9091	0.9856
		170	0.2407	0.1544	0.0066	0.0241	0.0074	0.0135	0.9332	0.9749	0.9697
		180	0.6879	0.5500	0.0168	0.0725	0.0240	0.0267	0.9024	0.9098	0.9949
BM	B + L acid	160	0.0817	0.0343	0.0122	0.0126	0.0159	0.0027	0.8782	0.9163	0.9685
		170	0.1329	0.0588	0.0416	0.0262	0.0221	0.0276	0.8756	0.8745	0.9657
		180	0.4333	0.2348	0.0574	0.0877	0.0574	0.0472	0.9970	0.9263	0.9727

For all substrates, the rate constants of *k*_1_ are greater compared to the rate constants of *k*_2_, indicating that Route 1 (Scheme 1) is the key reaction pathway in the alcoholysis reaction. This finding is consistent with our previous experimental observation.^15^ At the same time, the rate constants of *k*_3_ are far smaller than the rate constants of *k*_1_, suggesting that the generation of EL from EDGP is the rate-determining step and controls the overall reaction rate. The rate constants of *k*_1_ and *k*_2_ are larger for the ball milled sample, revealing that cellulose degradation can be promoted by mechanical ball milling. When using ball-milled corn stover as the substrate, the *k*_1_ and *k*_2_ values over single B acid SO_3_H-IL as catalyst are higher than using SO_3_H-IL and Al_2_(SO_4_)_3_. The results imply that SO_3_H-IL with stronger B acidity promotes cellulose degradation to generate EDGP and LGO. Meanwhile, *k*_3_ and *k*_4_ values increase when using SO_3_H-IL and Al_2_(SO_4_)_3_ as catalysts. The rate constant of EDGP and LGO conversion to EL rises after introducing Al^3+^ into the catalyst system, indicating a positive role in the formation of EL.

The kinetic rate constants are defined in terms of Arrhenius equation that combines the effects of reaction temperature:

10 *k* = *A* e^−*E*_a_/*RT*^

11
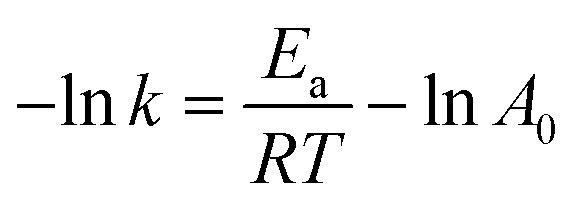
where *E*_a_ presents the activation energies, *A* is the pre-exponential factor of the main reaction, *R* is the determination coefficient, *T* is the reaction temperature.

The apparent activation energies were calculated from the plots of “−ln *k*” against “1/*RT*” with the data in Table 2. As shown in Table 2, the activation energies for cellulose decomposition to EDGP through alkylation (*E*_a1_) are lower than cellulose pyrolysis to LGO (*E*_a2_). Additionally, *E*_a3_ values for EDGP conversion to EL are lower than *E*_a4_ values for LGO decomposition to EL. The results confirmed that the alcoholysis of cellulose in corn stover tended to produce the key intermediate EDGP, and then EDGP generated the main product EL. Moreover, the activation energies (*E*_a1_ and *E*_a2_) of EDGP and LGO formation from ball-milled corn stover were significantly lower than those (*E*_a3_ and *E*_a4_) from nonball-milled sample, implying the positive effect of mechanical ball milling on cellulose degradation. Our group had previously proved that mechanical forces caused the depolymerization of macromolecules in the cell wall, thus improving the degradability of carbohydrate in biomass.^15,42^ The activation energy *E*_a1_ of cellulose decomposition to form EDGP catalyzed by the single B acid SO_3_H-IL is lower than that catalyzed by both SO_3_H-IL and Al_2_(SO_4_)_3_. This could be attributed to that pure B acid of SO_3_H-IL with strong B acidic is positive for Fischer glycosylation of cellulose,^11^ resulting in enhanced EDGP generation. Similar trend was observed for the *E*_a2_ of LGO formation from cellulose pyrolysis, which may be due to the fact that the strong B acidity in the pure SO_3_H-IL is beneficial for the dehydration reaction of cellulose. As a result, SO_3_H-IL with strong B acidity was essential for the dehydration reaction to produce LGO. The activation energy *E*_a3_ of EDGP to EL catalyzed by B acidic ionic liquid SO_3_H-IL and L acidic Al_2_(SO_4_)_3_ is significantly lower than catalyzed by single B acid SO_3_H-IL, indicating that B and L acid showed a synergistic effect on the formation of EL. Tao *et al.*^43^ reported that the introduction of Al^3+^ facilitated glucose-to-fructose isomerization, leading to the promoted conversion of cellulose. Based on the obtained parameters above, the kinetic model of cellulose alcoholysis is in line with experimental result.

Rate constants of cellulose decomposition with different raw materials and catalysts at various reaction temperatures

**Table:** 

Raw materia	Catalyst	*E*_a_					
		*E*_a1_	*E*_a2_	*E*_a3_	*E*_a4_	*E*_a5_	*E*_a6_
CM	B acid	157.51	183.30	128.53	137.48	213.80	180.36
BM	B acid	125.51	132.43	131.58	135.48	204.03	173.74
BM	B + L acid	135.57	156.38	104.21	126.88	233.87	158.10

**Table:** 

Raw materia	Catalyst	*R* ^2^					
		*R*_1_^2^	*R*_2_^2^	*R*_3_^2^	*R*_4_^2^	*R*_5_^2^	*R*_6_^2^
BM	B acid	0.974	0.998	0.985	0.996	0.991	0.981
BM	B acid	0.953	0.898	0.929	0.992	0.942	0.999
BM	B + L acid	0.939	0.933	0.920	0.906	0.893	0.976

## 4. Conclusion

The present research described a mild and efficient alcoholysis process for the synthesis of EL from ball milled corn stover catalyzed by B acidic ionic liquid SO_3_H-IL and L acidic Al_2_(SO_4_)_3_. The intermediate EDGP and LGO, together with the final product EL were main products from corn stover alcoholysis. The key reaction pathway in the alcoholysis reaction is the alkylation of cellulose to generate EDGP, followed by esterification to form EL. A simplified kinetic model of first-order reaction for EL formation from cellulose in corn stover was developed. A good fit between the kinetic model and experimental data was obtained. Mechanical ball milling promoted the conversion of cellulose to generate EDGP and LGO. Pure SO_3_H-IL gave high selectivity of EDGP with ball-milled corn stover and facilitated the LGO production. The combined SO_3_H-IL and Al_2_(SO_4_)_3_ catalysts substantially facilitated the formation of EL. The information about the kinetic parameter is of vital importance to discuss the effect of mechanical force and the combined B + L catalyst system on the alcoholysis, and the factors that can influence the performances of alcoholysis and conversion. Overall, this study will be of great help to accelerate the development of the industrial production of high value-added chemicals and fuels from lignocellulosic biomass.

## Conflicts of interest

The authors declare no competing financial interest.

## Supplementary Material
